# Three new species of eriophyoid mites (Acari, Eriophyoidea) associated with Lauraceae in China

**DOI:** 10.3897/zookeys.406.6897

**Published:** 2014-05-07

**Authors:** Qiong Wang, Xiao Han, Xiao-Feng Xue, Xiao-Yue Hong

**Affiliations:** 1 Department of Entomology, Nanjing Agricultural University, Nanjing, Jiangsu 210095, China

**Keywords:** Acari, plant feeding, Prostigmata, taxonomy

## Abstract

In this paper, three new species of eriophyoid mites in the family Eriophyidae associated with *Phoebe hunanensis* Hand.–Mazz. (Lauraceae), namely *Gammaphytoptus striatilobus* **sp. n.**, *Phyllocoptes setalsolenidion* **sp. n.**, and *Dechela phoebe* **sp. n.** are described and illustrated. All are vagrants causing no apparent damage to the same host plants.

## Introduction

Eriophyoidea is the lineage most highly adapted for plant feeding among the Acari. Among the vast array of eriophyoid taxa, patterns varying from narrow to extreme host specificity are far more prevalent, and repeatedly independent, than in other groups of phytophagous mites (Lindquist 1996a).

**Figure 1.**
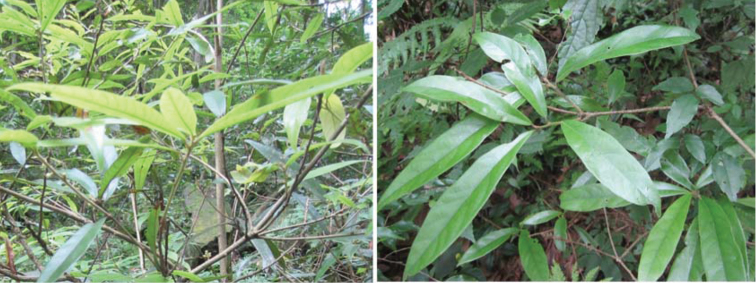
*Phoebe hunanensis* Hand.–Mazz. (Lauraceae) –The host plant in this study.

During July 2013, field surveys were conducted in Zhangjiajie National Forest Park of Hunan Province. We found three species from the same host *Phoebe hunanensis* Hand.–Mazz. (Lauraceae), this plant is native to South China naturally in the sheltered and moist places in valleys, under forests or by streams (Lee and Wei 1982). The host plant in this study is a shrub with the leaf blade lanceolate, and leaves close to leathery texture (Fig. 1).

So far, no eriophyoid mite species have been described or reported from *Phoebe hunanensis*. Two species are, however, known from other *Phoebe* species, which are *Bucculacus phoebus* Huang, 2001a and *Phyllocoptruta hungmaoensix* Xue, Cheng & Hong, 2012. Furthermore, seven of the nine recognized *Gammaphytoptus* species and three *Phyllocoptes* species are found associated with Lauraceae. A key to known *Gammaphytoptus* and *Phyllocoptes* species is given.

## Materials and methods

Eriophyoid mites were collected from plants with the aid of hand-lens (30×). Eriophyoids, together with their host plants, were placed in vials and stored in 75% ethanol. Each vial was marked with the following collection data: specimen number, date, host plant species name, colour of living mites, sample location, collector name and relationship of mite to the host plant. Collection data were also recorded in a notebook and examples of host plant parts were kept in a plant specimen folder in a dry environment for further identification and reference.

The morphological terminology follows Lindquist (1996b) and Amrine et al. (2003) and the generic classification was made according to Amrine et al. (2003). The liquid contents were pooled into a petri dish from the vials, then mite specimens were picked up using a fine pin and slide mounted using Keifer’s Booster and modified Berlese medium (Amrine and Manson 1996). Specimens were examined with the aid of a Zeiss A2 (Germany) research microscope equipped with phase contrast (A-plan phase objectives: ×10/0.25, ×20/0.45; EC plan-NEOFLUAR phase objectives: ×40/0.75; ×100/1.3 oil immersion) and schematic drawings were made. Images were taken with the same microscope (under 100× oil immersion with 10× eyepieces) using an Axio Cam MRc (Carl Zeiss) system, connected to a computer and using Axiovision image analysis software. Specimens were measured according to de Lillo et al. (2010). For each species, the holotype female measurement precedes the corresponding range for paratypes (given in parentheses). All measurements are in micrometres (μm) and are lengths when not otherwise specified. All type specimens are deposited as slide mounted specimens in the Arthropod/Mite Collection of the Department of Entomology, Nanjing Agricultural University (NJAU), Jiangsu Province, China.

## Results

### Family Eriophyidae Nalepa, 1898
Subfamily Cecidophyinae Keifer, 1966
Tribe Colomerini Newkirk & Keifer, 1975
Genus *Gammaphytoptus* Keifer, 1939

#### Gammaphytoptus striatilobus sp. n.

http://zoobank.org/F691F812-36E8-4CB7-BB9C-844634DE98CD

http://species-id.net/wiki/Gammaphytoptus_striatilobus

Figs 2 – 4

##### Description.

FEMALE: (n=11). Body fusiform, 187 (187–200), 61 (55–61) wide, 56 (56–60) thick; light yellow. **Gnathosoma** 24 (20–24), projecting obliquely downwards, pedipalp coxal setae (*ep*) 2 (2–3), dorsal pedipalp genual setae (*d*) 7 (6–7), cheliceral stylets 24 (23–24). **Prodorsal shield** 40 (39–42), 50 (48–50) wide, median, admedian and submedian lines complete and parallel, connected with transverse lines, shield design with anterior part covered with striaes; anterior shield lobe present 8 (8–9). Scapular tubercles on rear shield margin, 32 (32–33) apart, scapular setae (*sc*) 16 (15–16), projecting posteriorly. **Coxigenital region** with 4 (3–4) semiannuli between coxae and genitalia. Coxal plates with irregular lines, anterolateral setae on coxisternum I (*1b*) 7 (6–7), 12 (12–13) apart, proximal setae on coxisternum I (*1a*) 14 (14–17), 11 (11–12) apart, proximal setae on coxisternum II (*2a*) 26 (26–27), 26 (24–26) apart. Prosternal apodeme absent. **Leg I** 27 (26–27), femur 9 (9–10), basiventral femoral setae (*bv*) 8 (8–9); genu 5 (4–5), antaxial genual setae (*l*’’) 23 (21–23); tibia 6 (5–6), paraxial tibial setae (*l*’) 6 (6–7), located at centre; tarsus 5 (5–6), paraxia, fastigial, tarsal setae (*ft*’) 11 (11–12), antaxial, fastigial, tarsal setae (*ft*’’) 17 (17–18), paraxial, unguinal, tarsal setae (*u*’) 4 (4–5); tarsal empodium (*em*) 4 (4–5), simple, 7-rayed, tarsal solenidion (*ω*) 6 (6–7), rod-like. **Leg II** 24 (24–26), femur 9 (9–10), basiventral femoral setae (*bv*) 9 (8–9); genu 4 (4–5), antaxial genual setae (*l*’’) 7 (6–7); tibia 5 (5–6); tarsus 5 (5–6), paraxia, fastigial, tarsal setae (*ft*’) 5 (5–6), antaxial, fastigial, tarsal setae (*ft*’’) 17 (16–17), paraxial, unguinal, tarsal setae (*u*’) 3 (3–4); tarsal empodium (*em*) 5 (5–6), simple, 7-rayed, tarsal solenidion (*ω*) 8 (7–8), rod-like. **Opisthosoma** dorsally with 34 (34–35) semiannuli, with elliptical microtubercles, ventrally with 49 (49–51) semiannuli, with elliptical microtubercles. Setae *c2* 12 (12–13) on ventral semiannulus 8 (8–9), 48 (48–50) apart; setae *d* 41 (40–43) on ventral semiannulus 19 (18–19), 40 (37–40) apart; setae *e* 11 (11–14) on ventral semiannulus 30 (30–31), 22 (20–22) apart, setae *f* 25 (24–25) on 6th ventral semiannulus from rear, 18 (18–20) apart. Setae *h1* absent, *h2* 38 (37–38). **Female genitalia** 12 (12–15), 20 (20–22) wide, coverflap with two rows of ridges, the upper one with 14 (12–14) longitudinal ridges, the other with 12 (12–14) longitudinal ridges, setae *3a* 8 (7–8), 15 (14–15) apart.

MALE: (n=7, dorsal view). Body fusiform, 169–190, 56–63 wide; light yellow. **Gnathosoma** 19–22, projecting obliquely downwards, pedipalp coxal setae (*ep*) 2–3, dorsal pedipalp genual setae (*d*) 5–6, cheliceral stylets 23–24. **Prodorsal shield** 37–40, 47–50 wide, median, admedian and submedian lines complete and parallel, connected with transverse lines, shield design with anterior part covered with striaes; anterior shield lobe present 8–9. Scapular tubercles on rear shield margin, 27–30 apart, scapular setae (*sc*) 15–16, projecting posteriorly. **Coxigenital region** with 4–5 semiannuli between coxae and genitalia. Coxal plates with irregular lines, anterolateral setae on coxisternum I (*1b*) 5–6, 11–12 apart, proximal setae on coxisternum I (*1a*) 12–15, 8–11 apart, proximal setae on coxisternum II (*2a*) 25–26, 23–25 apart. Prosternal apodeme absent. **Leg I** 25–26, femur 9–10, basiventral femoral setae (*bv*) 8–9; genu 4–5, antaxial genual setae (*l*’’) 18–22; tibia 5–6, paraxial tibial setae (*l*’) 5–6, located at centre; tarsus 5–6, paraxia, fastigial, tarsal setae (*ft*’) 11–12, antaxial, fastigial, tarsal setae (*ft*’’) 15–17, paraxial, unguinal, tarsal setae (*u*’) 4–5; tarsal empodium (*em*) 4–5, simple, 7-rayed, tarsal solenidion (*ω*) 5–6, rod-like. **Leg II** 24–26, femur 9–10, basiventral femoral setae (*bv*) 8–10; genu 3–4, antaxial genual setae (*l*’’) 6–7; tibia 5–6; tarsus 5–6, paraxia, fastigial, tarsal setae (*ft*’) 5–6, antaxial, fastigial, tarsal setae (*ft*’’) 16–17, paraxial, unguinal, tarsal setae (*u*’) 3–4; tarsal empodium (*em*) 4–5, simple, 7-rayed, tarsal solenidion (*ω*) 6–7, rod-like. **Opisthosoma** dorsally with 33–37 semiannuli, with elliptical microtubercles, ventrally with 47–50 semiannuli, with elliptical microtubercles. Setae *c2* 15–16 on ventral semiannulus 8–9, 51–57 apart; setae *d* 40–41 on ventral semiannulus 17–19, 30–35 apart; setae *e* 13–15 on ventral semiannulus 29–33, 18–19 apart, setae *f* 24–25 on 6th ventral semiannulus from rear, 20–23 apart. Setae *h1* absent, *h2* 57–58. **Male genitalia** 16–18 wide, setae *3a* 7–8, 15–16 apart.

**Figure 2.**
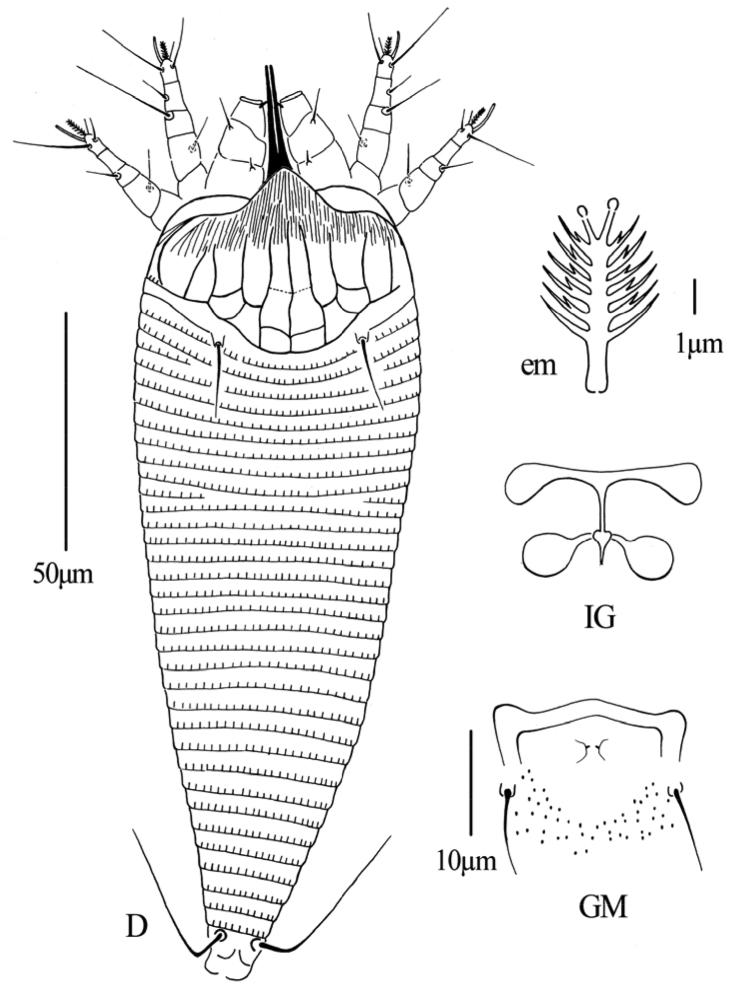
*Gammaphytoptus striatilobus* sp. n.: **D** dorsal view of female **em** empodium **IG** female internal genitalia **GM** male genital region.

**Figure 3.**
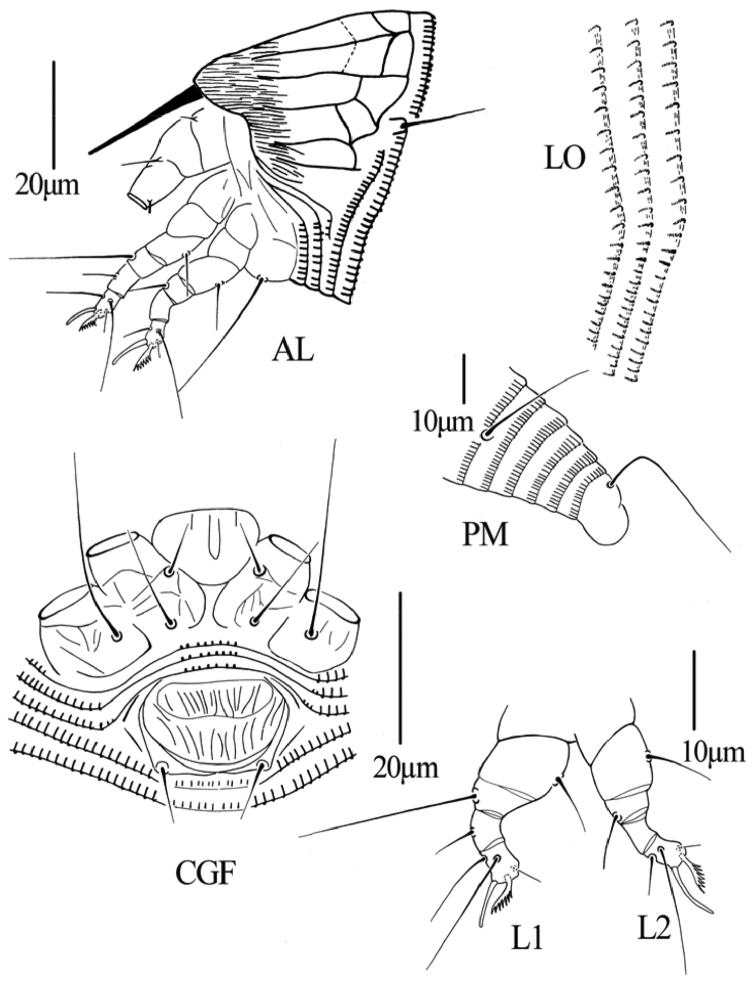
*Gammaphytoptus striatilobus* sp. n.: **AL** lateral view of anterior body region **LO** lateral view of annuli **PM** lateral view of posterior opisthosoma **CGF** female coxae and genitalia **L1** leg I **L2** leg II.

**Figure 4.**
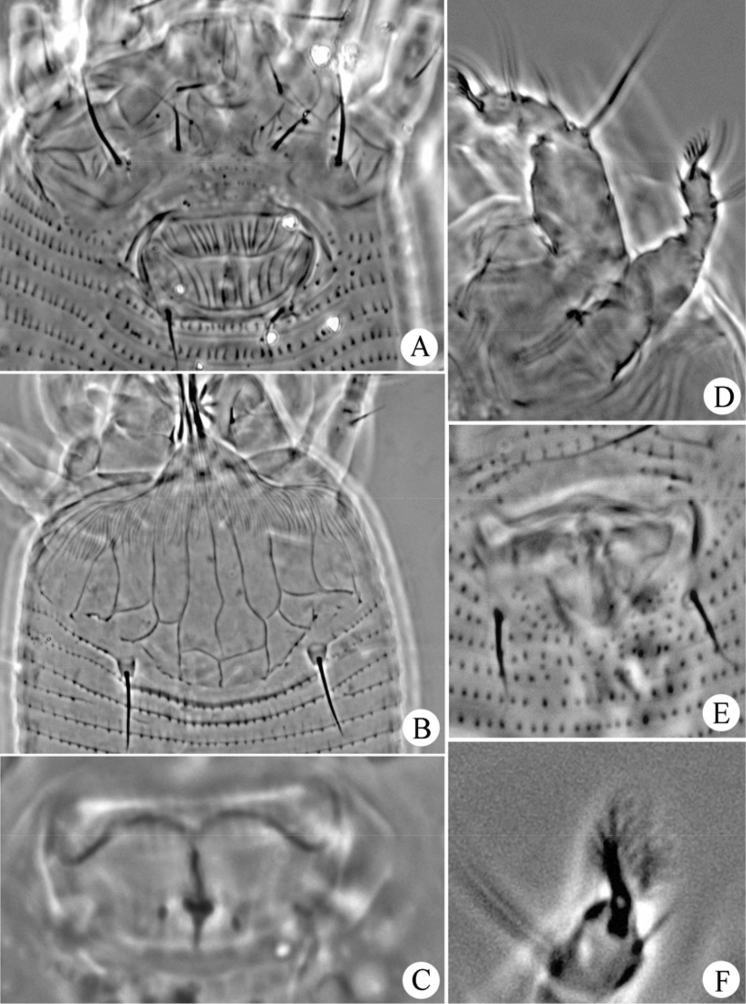
*Gammaphytoptus striatilobus* sp. n.: **A** coxae and female genitalia **B** prodorsal shield **C** female internal genitalia **D** leg I and leg II **E** male genitalia **F** empodium.

##### Type material.

**Holotype** female (slide number NJAUAcariEriHN128C.1; marked Holotype), from *Phoebe hunanensis* Hand.–Mazz. (Lauraceae), Zhangjiajie National Forest Park, Zhangjiajie City, Hunan Province, P.R. China, 29°20'41"N, 110°27'33"E, elevation 420m, 10 July 2013, coll. Qiong Wang, Xiao Han and Jingfeng Guo, deposited as a slide mounted specimen in the Arthropod/Mite Collection of the Department of Entomology, NJAU, Jiangsu Province, China. **Paratypes** 10 females and 7 males on 17 microscope slides (slide number NJAUAcariEriHN128C.2–128C.18), with the same data as holotype.

##### Relation to host.

This species is vagrant on lower part of the leaf surface. No damage to the host plant was observed.

##### Etymology.

The specific designation “striatilobus” is from the character of prodorsal shield lobe (“lobus”) marked with parellel fine impressed lines (“striatus”), masculine in gender.

##### Differential diagnosis.

This new species is similar to *Gammaphytoptus machilus* Li, Wei & Wang, 2009, but can be differentiated from the latter by having: the design of prodorsal shield with anterior part covered with striaes (a prodorsal shield design with two rows of cells in *Gammaphytoptus machilus*); dorsal semiannuli with elliptical microtubercles (dorsal semiannuli smooth in *Gammaphytoptus machilus*) and ventral semiannuli with elliptical microtubercles (ventral semiannuli with rounded microtubercles in *Gammaphytoptus machilus*).

### Family Eriophyidae Nalepa, 1898
Subfamily Phyllocoptinae Nalepa, 1892
Tribe Phyllocoptini Nalepa, 1892
Genus *Phyllocoptes* Nalepa, 1887

#### Phyllocoptes setalsolenidion sp. n.

http://zoobank.org/3A5DC24D-760D-4E86-A6F2-FA640D8F52E3

http://species-id.net/wiki/Phyllocoptes_setalsolenidion

Figs 5 – 7

##### Description.

FEMALE: (n=5, dorsal view). Body fusiform, 198 (186–198), 62 (59–65) wide; light yellow. **Gnathosoma** 29 (28–31), projecting obliquely downwards, pedipalp coxal setae (*ep*) 4 (4–5), dorsal pedipalp genual setae (*d*) 18 (18–21), cheliceral stylets 23 (20–24). **Prodorsal shield** 41 (41–42), 59 (59–60) wide, median, admedian and submedian lines absent, prodorsal shield with some short lines; anterior shield lobe 10 (10–14), acuminate, ending in a sharp point. Scapular tubercles 5 (5–6), ahead of rear shield margin, 19 (19–24) apart, scapular setae (*sc*) 10 (8–10), projecting centrad. **Coxigenital region** with 11 (10–11) semiannuli between coxae and genitalia. Coxal plates with fine granules, anterolateral setae on coxisternum I (*1b*) 15 (15–16), 12 (12–15) apart, proximal setae on coxisternum I (*1a*) 16 (15–16), 10 (10–11) apart, proximal setae on coxisternum II (*2a*) 33 (30–33), 25 (25–26) apart. Prosternal apodeme 4 (4–5). **Leg I** 37 (36–38), femur 12 (11–13), with fine granules, basiventral femoral setae (*bv*) 18 (18–20); genu 7 (6–7), antaxial genual setae (*l*’’) 22 (20–22); tibia 11 (10–11), paraxial tibial setae (*l*’) 10 (9–10), located at 1/3 from dorsal base; tarsus 7 (7–8), paraxia, fastigial, tarsal setae (*ft*’) 30 (29–30), antaxial, fastigial, tarsal setae (*ft*’’) 35 (32–35), paraxial, unguinal, tarsal setae (*u*’) 15 (15–17); tarsal empodium (*em*) 9 (8–9), simple, 8-rayed, tarsal solenidion (*ω*) 16 (16–17), seta-like. **Leg II** 31 (31–32), femur 10 (10–12), with fine granules, basiventral femoral setae (*bv*) 15 (15–16); genu 5 (4–5), antaxial genual setae (*l*’’) 14 (14–16); tibia 5 (5–6); tarsus 7 (5–7), paraxia, fastigial, tarsal setae (*ft*’) 18 (18–20), antaxial, fastigial, tarsal setae (*ft*’’) 28 (25–28), paraxial, unguinal, tarsal setae (*u*’) 14 (14–16); tarsal empodium (*em*) 10 (9–10), simple, 8-rayed, tarsal solenidion (*ω*) 15 (15–17), seta-like. **Opisthosoma** dorsally with 45 (45–48) semiannuli, smooth, ventrally with 70 (70–76) semiannuli, with small and rounded microtubercles set on rear annular margins, last 5th–6th semiannuli with elongated and linear tubercles. Setae *c2* 53 (53–55) on ventral semiannulus 14 (13–15), 49 (49–52) apart; setae *d* 59 (55–60) on ventral semiannulus 28 (26–28), 33 (32–33) apart; setae *e* 40 (39–42) on ventral semiannulus 42 (42–45), 15 (15–18) apart, setae *f* 21 (20–22) on 9th ventral semiannulus from rear, 17 (16–18) apart. Setae *h1* 4 (4–5), *h2* 25 (24–25). **Female genitalia** 14 (14–15), 26 (26–28) wide, coverflap with 14 (12–14) longitudinal ridges, setae *3a* 20 (17–20), 17 (17–19) apart.

MALE: (n=1, dorsal view). Body fusiform, 169, 54 wide; light yellow. **Gnathosoma** 27, projecting obliquely downwards, pedipalp coxal setae (*ep*) 4, dorsal pedipalp genual setae (*d*) 18, cheliceral stylets 22. **Prodorsal shield** 42, 57 wide, median, admedian and submedian lines absent, prodorsal shield with some short lines; anterior shield lobe 12, acuminate, ending in a sharp point. Scapular tubercles 5 ahead of rear shield margin, 24 apart, scapular setae (*sc*) 8, projecting centrad. **Coxigenital region** with 9 semiannuli between coxae and genitalia. Coxal plates with fine granules, anterolateral setae on coxisternum I (*1b*) 12, 14 apart, proximal setae on coxisternum I (*1a*) 17, 10 apart, proximal setae on coxisternum II (*2a*) 24, 25 apart. Prosternal apodeme 4. **Leg I** 30, femur 11, with fine granules, basiventral femoral setae (*bv*) 15; genu 4, antaxial genual setae (*l*’’) 20; tibia 7, paraxial tibial setae (*l*’) 10, located at 1/3 from dorsal base; tarsus 6, paraxia, fastigial, tarsal setae (*ft*’) 27, antaxial, fastigial, tarsal setae (*ft*’’) 28, paraxial, unguinal, tarsal setae (*u*’)14; tarsal empodium (*em*) 8, simple, 8-rayed, tarsal solenidion (*ω*) 15, seta-like. **Leg II** 26, femur 10, with fine granules, basiventral femoral setae (*bv*) 13; genu 4, antaxial genual setae (*l*’’) 14; tibia 5; tarsus 6, paraxia, fastigial, tarsal setae (*ft*’)13, antaxial, fastigial, tarsal setae (*ft*’’) 23, paraxial, unguinal, tarsal setae (*u*’) 11; tarsal empodium (*em*) 7, simple, 8-rayed, tarsal solenidion (*ω*) 14, seta-like. **Opisthosoma** dorsally with 42 semiannuli, smooth, ventrally with 71 semiannuli, with small and rounded microtubercles set on rear annular margins, last 5th–6th semiannuli with elongated and linear tubercles. Setae *c2* 50 on ventral semiannulus 14, 40 apart; setae *d* 52 on ventral semiannulus 25, 30 apart; setae *e* 40 on ventral semiannulus 43, 15 apart, setae *f* 24 on 9th ventral semiannulus from rear, 17 apart. Setae *h1* 5, *h2* 22. **Male genitalia** 21 wide, setae *3a* 11, 17 apart.

**Figure 5.**
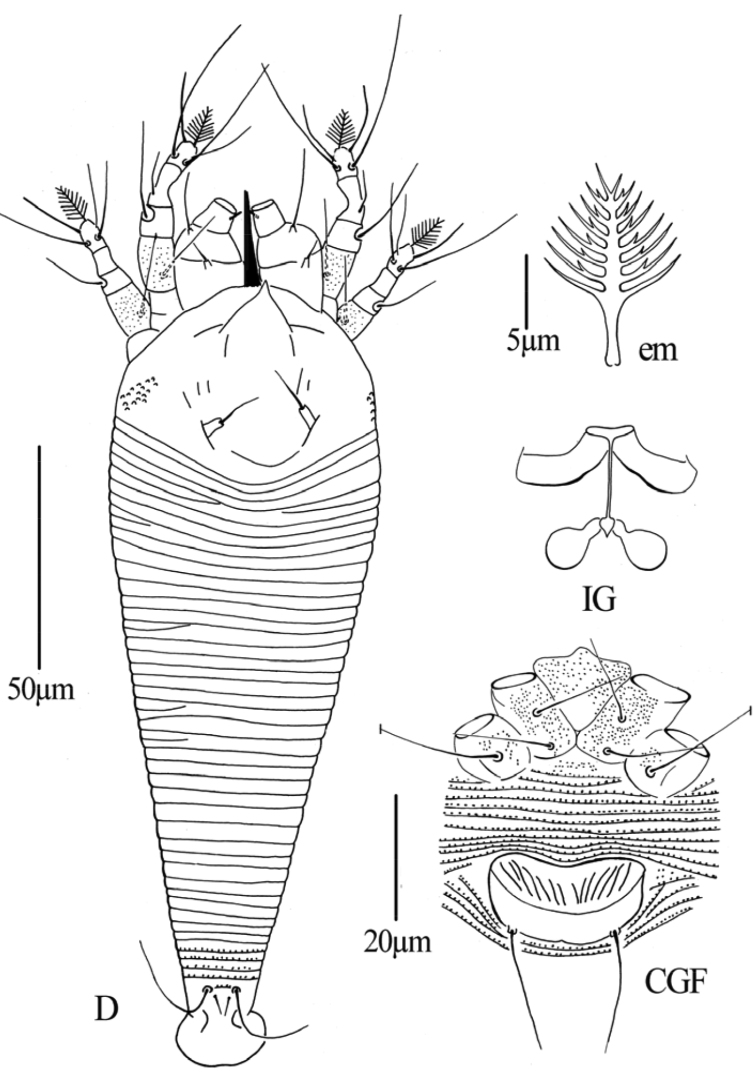
*Phyllocoptes setalsolenidion* sp. n.: **D** dorsal view of female **em** empodium **IG** female internal genitalia **CGF** female coxae and genitalia.

**Figure 6.**
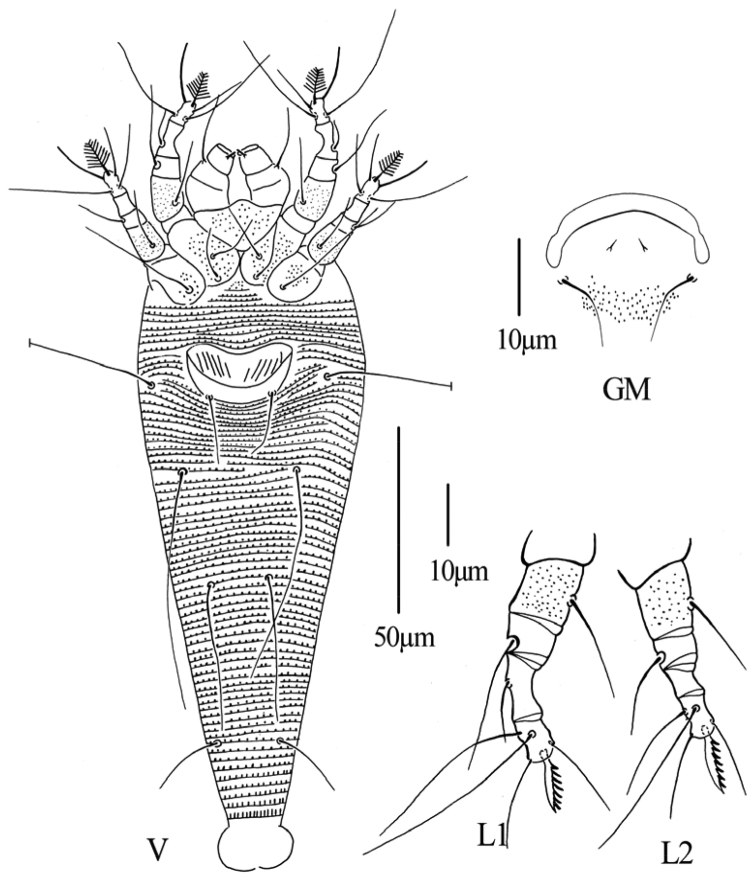
*Phyllocoptes setalsolenidion* sp. n.: **V** ventral view of female **GM** male genital region **L1** leg I **L2** leg II.

**Figure 7.**
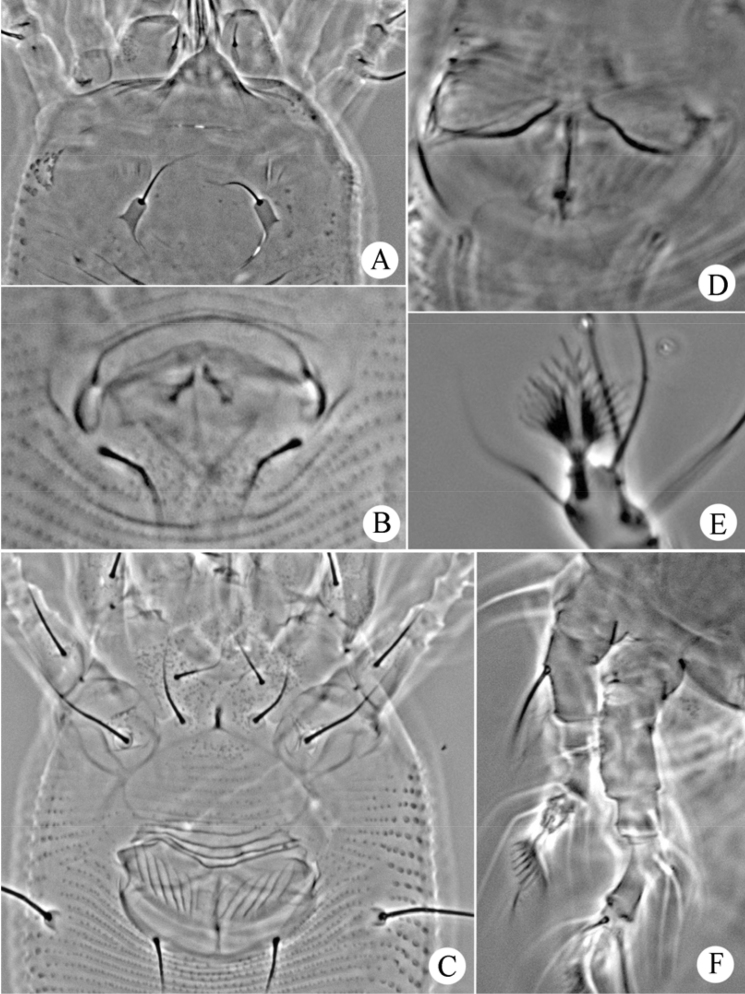
*Phyllocoptes setalsolenidion* sp. n.: **A** prodorsal shield **B** male genitalia **C** coxae and female genitalia **D** female internal genitalia **E** empodium **F** leg I and leg II.

##### Type material.

**Holotype** female (slide number NJAUAcariEriHN128A.1; marked Holotype), from *Phoebe hunanensis* Hand.–Mazz. (Lauraceae), Zhangjiajie National Forest Park, Zhangjiajie City, Hunan Province, P.R. China, 29°20'41"N, 110°27'33"E, elevation 420m, 10 July 2013, coll. Qiong Wang, Xiao Han and Jingfeng Guo, deposited as a slide mounted specimen in the Arthropod/Mite Collection of the Department of Entomology, NJAU, Jiangsu Province, China. **Paratypes** 4 females and 1 male on 5 microscope slides (slide number NJAUAcariEriHN128A.2-128A.6), with the same data as holotype.

##### Relation to host.

Vagrant on lower part of the leaf surface. No damage to the host plant was observed.

##### Etymology.

The specific designation *setalsolenidion* is derived from the shape (setal) of the tarsal solenidion. It is regarded as a noun phrase regardless of the gender and part of speech.

##### Differential diagnosis.

This new species is similar to *Phyllocoptes machilus* Wei, Xie & Chen, 2006, but can be differentiated from the latter mainly by possessing: prodorsal shield lacking median, admedian and submedian lines (with median line incomplete, present on the anterior and rear 1/5 respectively, admedian lines complete, forming a network in *Phyllocoptes machilus*); anterior shield lobe acuminate, ending in a sharp point (with small frontal lobe in *Phyllocoptes machilus*); femur having fine granules (femur smooth in *Phyllocoptes machilus*) and tarsal empodium 8-rayed, tarsal solenidion seta-like (tarsal empodium 4-rayed, tarsal solenidion knobbed).

### Family Eriophyidae Nalepa, 1898
Subfamily Cecidophyinae Keifer, 1966
Tribe Cecidophyini Keifer, 1966
Genus *Dechela* Keifer, 1965

#### Dechela phoebe sp. n.

http://zoobank.org/E018B236-5BB5-485C-A921-66AC407D15A8

http://species-id.net/wiki/Dechela_phoebe

Figs 8 – 10

##### Description.

FEMALE: (n=13). Body vermiform, 187 (183–192), 60 (55–60) wide, 62 (57–62) thick; light yellow. **Gnathosoma** 15 (15–18), projecting obliquely downwards, pedipalp coxal setae (*ep*) 3 (2–3), dorsal pedipalp genual setae (*d*) 4 (4–5), cheliceral stylets 12 (12–14). **Prodorsal shield** 27 (26–30), 51 (45–51) wide, covered with short lines; anterior shield lobe absent. Scapular tubercles and scapular setae absent. **Coxigenital region** with 2 (2–3) indistinct semiannuli between coxae and genitalia. Coxal plates with minute lines, anterolateral setae on coxisternum I (*1b*) absent, proximal setae on coxisternum I (*1a*) 12 (12–15), 13 (11–13) apart, proximal setae on coxisternum II (*2a*) 19 (18–21), 28 (27–29) apart. Prosternal apodeme absent. **Leg I** 21 (20–22), femur 6 (6–7), with some dash lines on ventral part, basiventral femoral setae (*bv*) 9 (9–11); genu 4 (3–4), antaxial genual setae (*l*’’) 24 (22–24); tibia 3 (2–3), paraxial tibial setae (*l*’) absent; tarsus 5 (5–6), paraxia, fastigial, tarsal setae (*ft*’) 13 (13–15), antaxial, fastigial, tarsal setae (*ft*’’) 17 (16–18), paraxial, unguinal, tarsal setae (*u*’) 5 (5–7); tarsal empodium (*em*) 7 (7–8), simple, 7-rayed outside, 5-rayed inside, tarsal solenidion (*ω*) 5 (5–6), rod-like, located below empodia. **Leg II** 18 (18–19), femur 6 (5–6), with some dash lines on ventral part, basiventral femoral setae (*bv*) 10 (10–11); genu 4 (3–4), antaxial genual setae (*l*’’) absent; tibia 2 (2–3); tarsus 6 (5–6), paraxia, fastigial, tarsal setae (*ft*’) 7 (7–8), antaxial, fastigial, tarsal setae (*ft*’’) 18 (18–23), paraxial, unguinal, tarsal setae (*u*’) 5 (4–5); tarsal empodium (*em*) 6 (6–7), simple, 7-rayed outside, 5-rayed inside, tarsal solenidion (*ω*) 15 (15–16), rod-like. **Opisthosoma** dorsally with 55 (55–57) annuli, with elliptical microtubercles, ventrally with 56 (56–58) annuli, with elliptical microtubercles. Setae *c2* 10 (10–11) on ventral annulus 8 (7–9), 48 (48–50) apart; setae *d* 53 (50–55) on ventral annulus 16 (16–18), 38 (38–40) apart; setae *e* 50 (50–52) on ventral annulus 32 (31–32), 26 (26–27) apart, setae *f* 15 (15–16) on 6th ventral annulus from rear, 12 (11–12) apart. Setae *h1* absent, *h2* 21 (20–23). **Female genitalia** 12 (12–14), 19 (18–19) wide, coverflap with transverse dashes, setae *3a* 30 (27–30), 16 (15–16) apart.

MALE: (n=2, dorsal view). Body vermiform, 175–192, 48–54 wide; light yellow. **Gnathosoma** 20–21, projecting obliquely downwards, pedipalp coxal setae (*ep*) 2–3, dorsal pedipalp genual setae (*d*) 4–5, cheliceral stylets 10–13. **Prodorsal shield** 25–27, 40–50 wide, covered with short lines; anterior shield lobe absent. Scapular tubercles and scapular setae absent. **Coxigenital region** with 2–3 indistinct semiannuli between coxae and genitalia. Coxal plates with minute lines, anterolateral setae on coxisternum I (*1b*) absent, proximal setae on coxisternum I (*1a*) 12–13, 9–10 apart, proximal setae on coxisternum II (*2a*) 17–20, 24–25 apart. Prosternal apodeme absent. **Leg I** 17–20, femur 6–7, with some dash lines on ventral part, basiventral femoral setae (*bv*) 8–9; genu 3–4, antaxial genual setae (*l*’’) 20–21; tibia 3–4, paraxial tibial setae (*l*’) absent; tarsus 4–5, paraxia, fastigial, tarsal setae (*ft*’) 10–11, antaxial, fastigial, tarsal setae (*ft*’’) 14–16, paraxial, unguinal, tarsal setae (*u*’) 5–6; tarsal empodium (*em*) 6–7, simple, 7-rayed outside, 5-rayed inside, tarsal solenidion (*ω*) 5–6, rod-like, located below empodia. **Leg II** 17–20, femur 5–6, with some dash lines on ventral part, basiventral femoral setae (*bv*) 8–9; genu 2–3, antaxial genual setae (*l*’’) absent; tibia 2–3; tarsus 5–6, paraxia, fastigial, tarsal setae (*ft*’) 6–7, antaxial, fastigial, tarsal setae (*ft*’’) 18–19, paraxial, unguinal, tarsal setae (*u*’) 4–5; tarsal empodium (*em*) 5–6, simple, 7-rayed outside, 5-rayed inside, tarsal solenidion (*ω*) 13–15, rod-like. **Opisthosoma** dorsally with 54–56 annuli, with elliptical microtubercles, ventrally with 56–57 annuli, with elliptical microtubercles. Setae *c2* 15–16 on ventral annulus 8–9, 40–41 apart; setae *d* 43–45 on ventral annulus 16–17, 30–34 apart; setae *e* 43–44 on ventral annulus 30–32, 23–24 apart, setae *f* 15–16 on 6th ventral annulus from rear, 10–11 apart. Setae *h1* absent, *h2* 26–27. **Male genitalia** 18–19 wide, setae *3a* 26–30, 15–16 apart.

**Figure 8.**
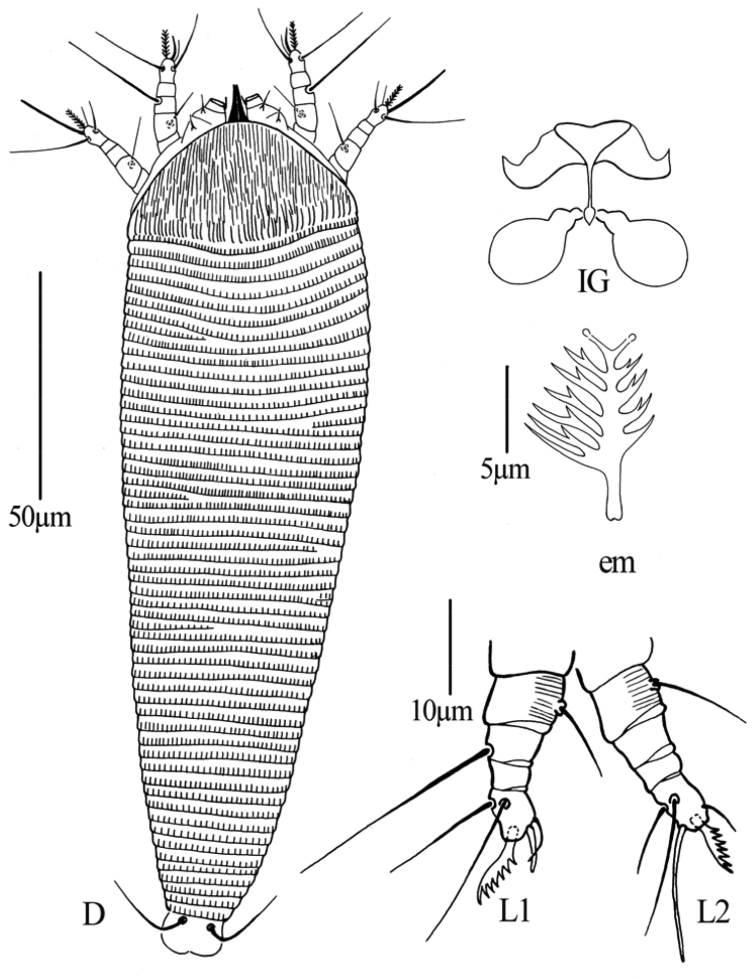
*Dechela phoebe* sp. n.: **D** dorsal view of female **IG** female internal genitalia **em** empodium **L1** Leg I **L2** leg II.

**Figure 9.**
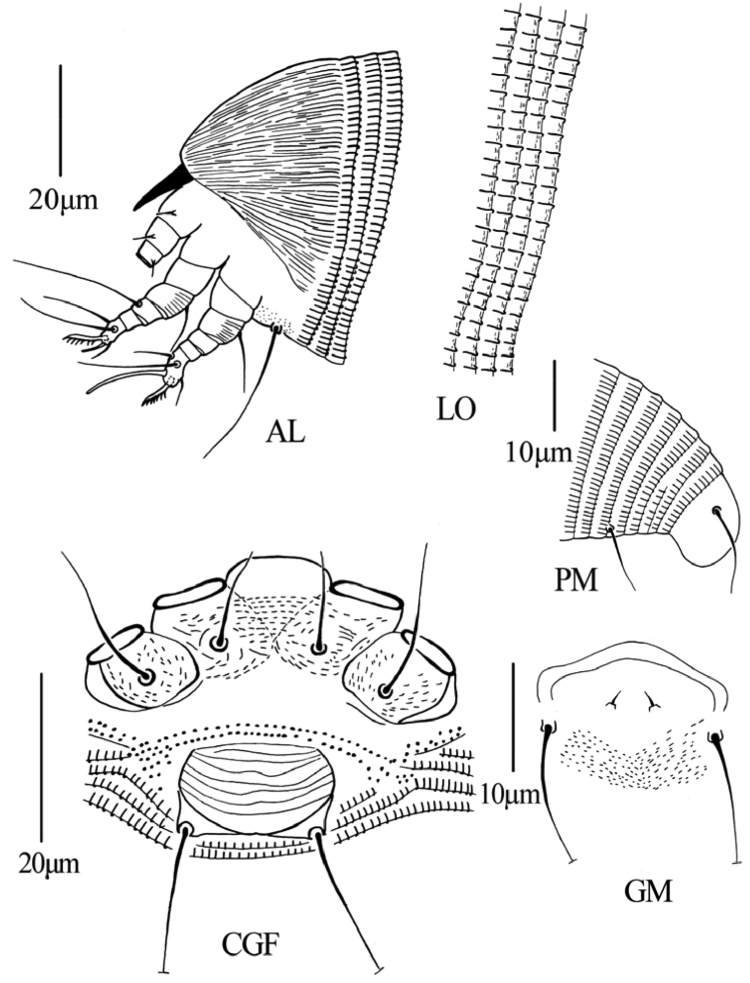
*Dechela phoebe* sp. n.: **AL** lateral view of anterior body **LO** lateral view of annuli **PM** lateral view of posterior opisthosoma **CGF** female coxae and genitalia **GM** male genital region.

**Figure 10.**
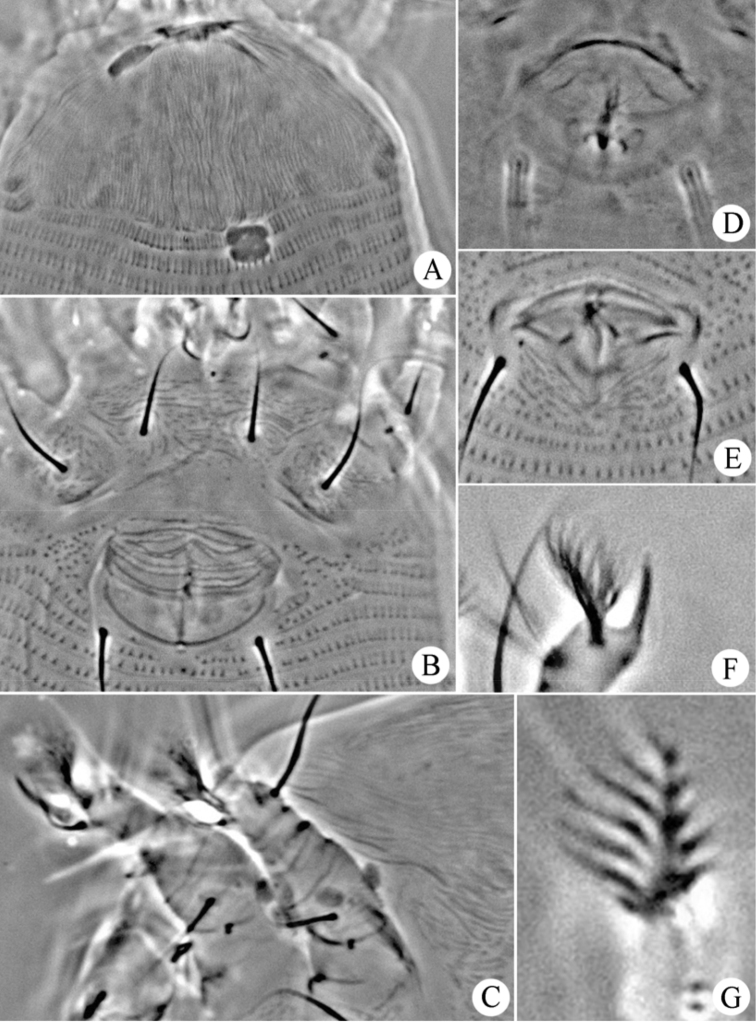
*Dechela phoebe* sp. n.: **A** prodorsal shield **B** coxae and female genitalia **C** leg I and leg II **D** female internal genitalia **E** male genitalia **F** tarsal solenidion of leg I **G** empodium.

##### Material examined.

13 females and 2 males on 15 microscope slides (slide number NJAUAcariEriHN128B.1-128B.15), from *Phoebe hunanensis* Hand.–Mazz. (Lauraceae), Zhangjiajie National Forest Park, Zhangjiajie City, Hunan Province, P.R. China, 29°20'41"N, 110°27'33"E, elevation 420m, 10 July 2013, coll. Qiong Wang, Xiao Han and Jingfeng Guo, deposited as a slide mounted specimen in the Arthropod/Mite Collection of the Department of Entomology, NJAU, Jiangsu Province, China.

##### Relation to host.

Vagrant on lower part of the leaf surface. No damage to the host plant was observed.

##### Etymology.

The specific designation *Phoebe* is derived from the generic name of the host plant; feminine in gender.

##### Differential diagnosis.

This new species is very similar to *Dechela epelis* Keifer, 1965, but some quantitative characters can be used to separate them (Table 1).

**Table 1.** The differential diagnosis between *Dechela epelis*, Keifer and *Dechela phoebe* sp. n.

Characters	*Dechela epelis* Keifer	*Dechela phoebe* sp. n.
body length	175–190	187 (183–192)
body width	42–45	60 (55–60)
gnathosoma length	19	15 (15–18)
*d*	3.5	4 (4–5)
shield length	26	27 (26–30)
shield width	32	51 (45–51)
anterior shield lobe	present	absent
coxisternal area	coxae with curved lines of granules or short dashes	coxal plates with minute lines
leg I	20–21	21 (20–22)
tibia I/*l*’	3/absent	3 (2–3)/absent
tarsus I/*ω*	5, tarsal solenidion 4 straight or slightly curved laterally	5 (5–6), tarsal solenidion 5 (5–6), slightly curved laterally
em I	7-rayed on outside, 5-rayed inside	7 (7–8), 7-rayed on outside, 5-rayed inside
leg II	20	18 (18–19)
tibia II	2	2 (2–3)
tarsus II/*ω*	5, tarsal solenidion 10 straight	6 (5–6), tarsal solenidion 15 (15–16) straight
em II	7-rayed on outside, 5-rayed inside	6 (6–7), 7-rayed on outside, 5-rayed inside
dorsal annuli	62	55 (55–57)
ventral annuli	62	56 (56–58)
*c2*	15, on 6–8 annuli behind shield, projecting up and forward	10 (10–11), on 8 (7–9) annuli from coxae
*d*	36, on 19 annuli	53 (50–55), on 16 (16–18) annuli
*e*	42, on 37 annuli	50 (50–52),on 32 (31–32) annuli
*f*	14, on 4–5 from rear	15 (15–16), on 6 from rear
*h1*	absent	absent
female genitalia/*3a*	16 wide, 11 long; coverflap with transverse and gently curved lines of granules and dashes; 13 long	19 (18–19) wide, 12 (12–14) long; coverflap with transverse dashes; 30 (27–30) long
host plant	*Bixa* sp. (Bixaceae)	*Phoebe hunanensis* Hand.–Mazz. (Lauraceae)

### A key to *Gammaphytoptus*, *Phyllocoptes* species known from Lauraceae

**Table:** 

1	Female genitalia appressed to coxae, ridges on female coverflap in two uneven ranks	2
–	Female genitalia not appressed to coxae, ridges on female coverflap in one rank	9
2	The anterior part of prodorsal shield design covered with striaes	*Gammaphytoptus striatilobus* sp. n.
–	Prodorsal shield design without short lines	3
3	Dorsal annuli smooth	*Gammaphytoptus machilus* Li, Wei & Wang, 2009
–	Dorsal annuli with microtubercles	4
4	Empodia 6-rayed or 7-rayed	5
–	Empodia 5-rayed	6
5	Empodia 6-rayed, prodorsal shield pattern of part longitudinal and part network lines	*Gammaphytoptus camphorae* Keifer, 1939
–	Empodia 7-rayed, prodorsal shield without median line and submedian, admedian lines complete	*Gammaphytoptus commune* Huang & Wang, 2009
6	Prodorsal shield design with median line complete	7
–	Prodorsal shield design with median line incomplete	8
7	Prodorsal shield design complex and anteriorly with a number of cells	*Gammaphytoptus bengalensis* Das & Chakrabarti, 1985
–	Prodorsal shield design simple and with a number of longitudinal parallel lines	*Gammaphytoptus litseasis* Ghosh & Chakrabarti, 1982
8	Setae *h1* present	*Gammaphytoptus zuihoensus* Huang & Wang, 2004
–	Setae *h1* absent	*Gammaphytoptus litseaus* Huang, 2001b
9	Dosal annuli smooth	*Phyllocoptes setalsolenidion* sp. n.
–	Dosal annuli with microtubercles	10
10	Empodia 4-rayed	*Phyllocoptes machilus* Wei, Xie & Chen, 2006
–	Empodia 5-rayed	11
11	Coxae with short curved lines and dashes	*Phyllocoptes linderafolius* Styer, 1975
–	Coxae with granular lines	*Phyllocoptes sassafrasella* Keifer, 1959

## Supplementary Material

XML Treatment for Gammaphytoptus striatilobus

XML Treatment for Phyllocoptes setalsolenidion

XML Treatment for Dechela phoebe
